# Supplementary material for: Hepatitis C Virus among Female Sex Workers: A Cross-Sectional Study Conducted along Rivers and Highways in the Amazon Region
Source: Pathogens. 2019 Nov 14;8(4):236. doi: 10.3390/pathogens8040236 (PMC6963267; doi:10.3390/pathogens8040236)
Supplement: Supplementary file 1 [file pathogens-08-00236-s001.pdf]

SUPPLEMENTARY MATERIALS

Title: Hepatitis C virus among female sex workers: a cross-sectional study conducted by rivers and highways in the Amazon region.

Authors: Aldemir B. Oliveira-Filho, Diego Wendel F. Aires, Natalia dos Santos Cavalcante, Nairis Costa Raiol, Brenda Luena A. Lisboa, Evelen da Cruz Coelho, Paula Cristina R. Frade, Luana Mota da Costa, Luiz Marcelo L. Pinheiro, Luiz Fernando A. Machado, Luisa Caricio Martins, Gláucia C. Silva-Oliveira, João Renato R. Pinho, Emil Kupek, and José Alexandre R. Lemos.

Table S1. Information on the collection of biological samples and personal data from the female sex workers in the Amazon region.

Location	Type	Main access	Methods[1]	Sample size
Breves	Town	Parauau River	TLS, RDS	30
Bagre	Town	Jacundá River	TLS	11
Curralinho	Town	Pará River	RDS	13
Melgaço	Town	Tajapurú River	RDS	13
São Sebastião da Boa Vista	Town	Boa Vista River	RDS	12
Antônio Lemos	Community	Tajapurú River	TAS	7
Capinal	Community	Tajapurú River	TAS	5
São Francisco	Community	Tajapurú River	TAS	5
Ramex	Community	Tajapurú River	TAS	5
São Sebastião	Community	Tajapurú River	TLS	4
Nossa Senhora de Fátima	Community	Tajapurú River	TLS	3
Mainard	Community	Jaburú River	TLS	3
Intel	Community	Mearin River	TLS	3
Campo Beija Flor	Community	Tajapurú River	TLS	3
Zé Gama	Community	Pracaxi River	TLS	2
Nova Canaã	Community	Pracaxi River	TLS	3
Santa Cruz	Community	Pracaxi River	TLS	3
Monte Tabu	Community	Parauau River	TLS	4
São José	Community	Parauau River	TLS	3
Corcovado	Community	Parauau River	TLS	4
Magebras	Community	Parauau River	TAS	5
Bom Jesus	Community	Aturiá River	TAS	6
Jupatituba	Community	Parauau River	TAS	6
Soure	Town	Paracauari River	TAS	15
Salvaterra	Town	Paracauari River	TAS	12
Ananindeua	Town	BR-316 Highway	RDS	32
Marituba	Town	BR-316 Highway	RDS	29
Santa Izabel do Pará	Town	BR-316 Highway	RDS	21
Vigia	Town	PA-010 Highway	RDS	13
Castanhal	Town	BR-316 Highway	RDS	34
Terra Alta	Town	PA-136 Highway	COS	7
Marapanim[2]	Town	PA-136 and PA-138	RDS	14
Santa Maria do Pará	Town	BR-316 and BR-010	RDS	24
Capanema	Town	BR-316 Highway	RDS	37
São Miguel do Guamá	Town	BR-010 Highway	RDS	11
Salinópolis	Town	PA-124 Highway	COS	10

[1]Sampling Methods: Respondent-driven sampling (RDS), Time location sampling (TLS), Take-all sampling (TAS), Convenience sampling (COS). [2] Only in Marudá District.

Table S2. Bivariate and multivariate analysis of factors not associated with HCV exposure among FSWs in the Amazon region.

**Factors**	**Total**	**HCV +**	**Bivariate OR (95% CI)**	**Multivariate OR (95% CI)**
27+ years *versus* (*vs.*) 18-26 years	170	20	1.2 (0.7 – 2.3)	1.1 (0.8 - 2.2)
Non-white *vs*. White	307	32	0.9 (0.5 – 1.8)	0.8 (0.4 - 2.0)
Born in the state of Pará *vs.* Not born in the state of Pará	318	38	2.0 (0.8 – 4.9)	1.8 (0.7 - 3.9)
Heterosexual *vs*. Same-sex (including bisexual)	381	40	1.1 (0.4 – 3.3)	1.2 (0.5 - 3.2)
Single, separated or widowed *vs*. Married or co-habitating *	379	39	0.6 (0.2 – 1.8)	0.8 (0.3 - 1.5)
Blood transfusion received *vs*. Did not receive blood transfusion	46	6	1.7 (0.7 – 4.5)	1.5 (0.8 - 4.0)
Had surgery *vs*. Did not have surgery	63	7	1.1 (0.5 – 2.5)	0.8 (0.3 - 2.2)
Performed invasive dental treatment *vs*. No invasive dental treatment	136	12	0.7 (0.3 – 1.5)	0.6 (0.3 - 1.7)
Tattoos *vs*. No tattoos	210	24	1.2 (0.6 – 2.2)	1.4 (0.5 - 2.1)
Condom exemption for regular clients *vs*. Condom use with regular clients **	76	6	0.7 (0.3 – 1.8)	0.6 (0.4 - 2.1)

**Last 12 months; **Last 7 days.*

Table S3. Association between epidemiological factors and HCV genotypes in female sex workers in the Amazon region (bivariate analysis).

Factors	cDNA HCV+	Genotypes		*p-value****
		**1 (%)**	**3 (%)**	
Total	32	26 (81.2)	6 (18.8)	-
Age (years)				
18 – 26	20	18 (90.0)	2 (10.0)	0.10
≥ 27	12	8 (67.0)	4 (33.0)	
Colour/Race (self-declaration)				
Non-White	26	22 (84.6)	4 (15.4)	0.31
White	6	4 (67.0)	2 (33.0)	
Origin				
Born in the state of Pará	29	24 (82.8)	5 (17.2)	
Not born in the state of Pará	3	2 (67.0)	1 (33.0)	0.49
Sexual orientation				
Heterosexual	30	25 (83.3)	5 (16.7)	0.24
Same-sex (including bisexual)	2	1 (50.0)	1 (50.0)	
Education Level				
Up to elementary school	29	24 (82.8)	5 (17.2)	0.49
High school or more	3	2 (67.0)	1 (33.0)	
Marital status*				
Single, separated or widowed	30	25 (83.3)	5 (17.2)	0.24
Married or co-habitating	2	1 (50.0)	1 (50.0)	
Monthly income (minimum wage)*				
Up to 1	29	24 (82.8)	5 (17.2)	0.49
≥ 2	3	2 (67.0)	1 (33.0)	
Blood transfusion				
Yes	2	1 (50.0)	1 (50.0)	0.24
No	30	25 (83.3)	5 (17.2)	
Surgery				
Yes	2	1 (50.0)	1 (50.0)	0.24
No	30	25 (83.3)	5 (17.2)	
Invasive dental treatment				
Yes	4	3 (75.0)	1 (25.0)	0.73
No	28	23 (82.1)	5 (17.9)	
Tattoos				
Yes	28	23 (82.1)	5 (17.9)	0.73
No	4	3 (75.0)	1 (25.0)	
Illicit drug use (injectable or inhaled)*				
Yes	27	22 (81.5)	5 (18.5)	0.94
No	5	4 (80.0)	1 (20.0)	
Unprotected sex**				
Yes	30	25 (83.3)	5 (16.7)	0.24
No	2	1 (50.0)	1 (50.0)	
More than five sexual partners**				
Yes	24	20 (83.3)	4 (16.7)	0.60
No	8	6 (75.0)	2 (25.0)	
Condom exemption for clients paying extra**				
Yes	27	22 (81.5)	5 (18.5)	0.94
No	5	4 (80.0)	1 (20.0)	
Condom exemption for regular clients**				
Yes	3	2 (67.0)	1 (33.0)	0.49
No	29	24 (82.8)	5 (17.2)	

More than seven years working in the sex trade				
Yes	28	23 (82.1)	5 (17.9)	0.73
No	4	3 (67.0)	1 (33.0)	
Changes in genitalia*				
Yes	30	25 (83.3)	5 (16.7)	0.24
No	2	1 (50.0)	1 (50.0)	
Medical/gynecological examination*				
No	7	5 (71.4)	2 (28.6)	0.45
Yes	25	21 (84.0)	4 (16.0)	

Last 12 months; **Last 7 days; ; * Chi-square test.*

Table S4. Amino acid substitutions and frequency of female sex workers with substations conferring probable resistance to protease inhibitors.

Sample	Subtype	Amino acid substitutions	Probable resistance
FSW01	1a	N174S	Telaprevir
FSW02	1a	N174S	Telaprevir
FSW03	1a	N174S	Telaprevir
FSW04	1a	N174S	Telaprevir
FSW05	1a	-	None
FSW06	1a	-	None
FSW07	1a	N174S	Telaprevir
FSW08	1a	N174S	Telaprevir
FSW09	1a	-	None
FSW10	1a	-	None
FSW11	1a	-	None
FSW12	1b	-	None
FSW13	1b	Y56F	Grazoprevir
FSW14	1b	-	None
FSW15	1b	-	None
FSW16	1b	-	None
FSW17	1b	S122G	Grazoprevir
FSW18	1b	-	None
FSW20	1b	S122G	Grazoprevir
FSW21	1b	-	None
FSW22	1b	-	None
FSW23	1b	-	None
FSW24	1b	-	None
FSW25	1b	S122G	Grazoprevir
FSW26	1b	S122G	Grazoprevir
FSW27	1b	S122G	Grazoprevir
**Total frequency (**(N174S, Y56F or S122G**)**			**12/26 (46.2%)**
Telaprevir (N174S)			6/26 (23.1%)
Grazoprevir (Y56F or S122G)			6/26 (23.1%)

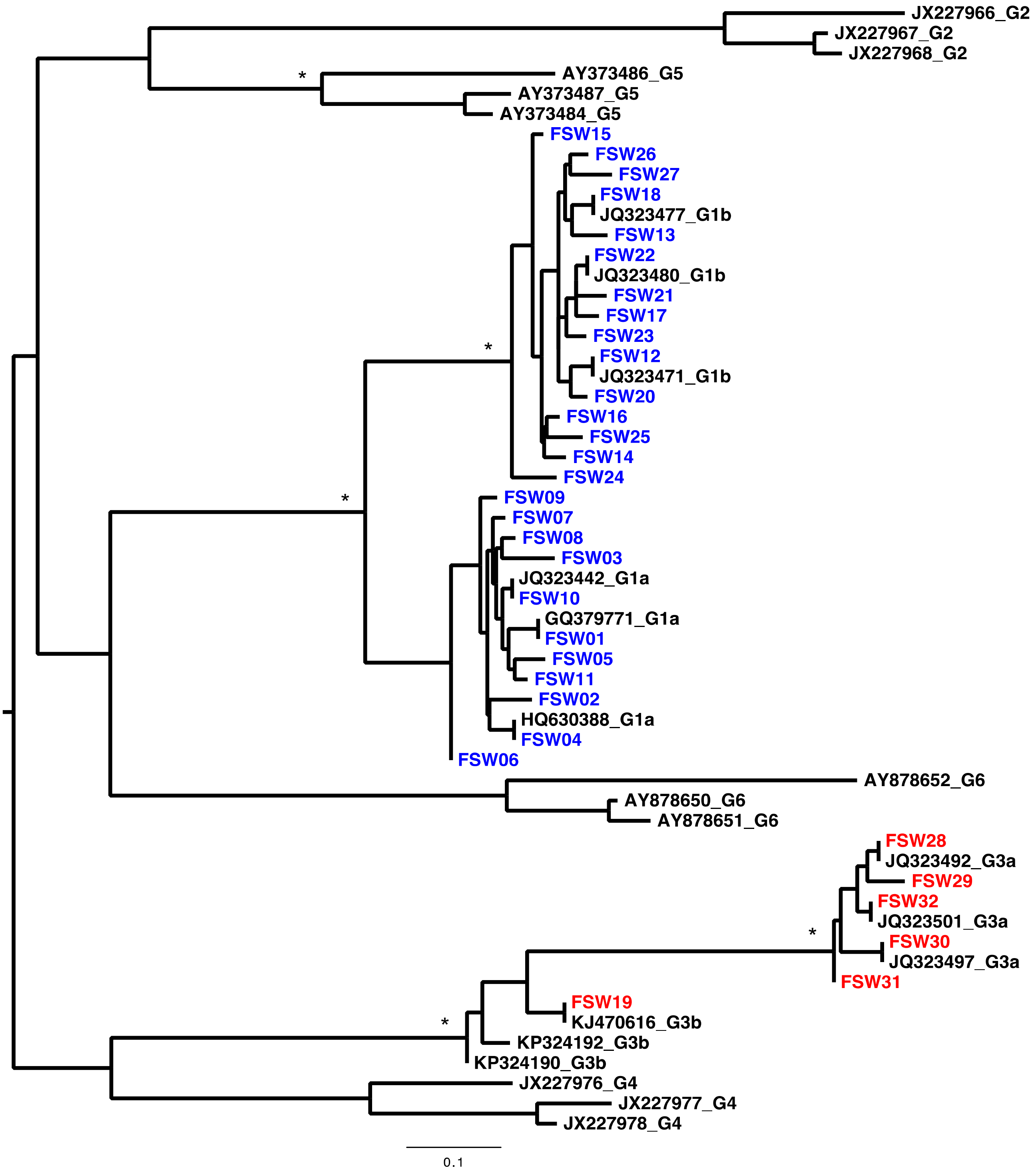

Figure S1: Maximum likelihood phylogeny of HCV NS5B gene sequences isolated in 32 female sex workers in the Amazon region. The tree was rooted at the midpoint. Asterisks point to key nodes with high support (aLRT $\geq$ 0.90). Samples of this study can be identified by the acronym FSW + number. Samples of FSWs belonging to genotypes 1 and 3 are highlighted in blue and red, respectively.
